# Recovery from Heart Failure

**Published:** 1917-03

**Authors:** 


### RECOVERY FROM HEART FAILURE.

An instance of remarkable recovery from heart failure is reported in *Guy's Hospital Gazette,* October 21st. A six-year-old boy had been given an anesthetic (chloroform two, ether three parts; on open mask) for the removal of tonsils and adenoids, and the operation had been more than half completed when an increasing cyanosis was observed, and the breathing stopped altogether. Artificial respiration was tried without result, and the pulse could not be felt at the wrist. Various remedial measures were promptly applied; oxygen was given, and injections of brandy, ether, and of pituitrin were tried in succession, but failed to produce any signs of restoration.

When about twelve minutes had elapsed since the cessation of respiration, it was decided to open the abdomen and massage the heart. This was effected by a hand insinuated between the liver and diaphragm, and at first no improvement followed vigorous massage. The massage was continued along with artificial respiration, and "an occasional sighing respiration took place." The report states that after about twenty more squeezes, suddenly the heart was felt to begin beating and the respiration became reestablished. Further recovery was very slow, but eventually complete. From the careful estimates of those present, it was considered certain that the heart had stopped at least thirteen minutes.—*The Dental Record.*

